# A Microbiological Assay of Common Operating Room (OR) Tapes: Developing a Culture for Patient Safety

**DOI:** 10.7759/cureus.31919

**Published:** 2022-11-26

**Authors:** Gaurav Chauhan, Aman Upadhyay, Samvid Dwivedi, Robert J Tibbetts, Suresh K Srinivasan

**Affiliations:** 1 Anesthesiology and Perioperative Medicine, University of Pittsburgh Medical Center Presbyterian, Pennsylvania, USA; 2 Pain Medicine, McLaren Oakland Hospital, Pontiac, USA; 3 Anesthesiology, Henry Ford Health System, Detroit, USA; 4 Pathology, Henry Ford Health System, Detroit, USA; 5 Pain Management, Trinity West Medical Center, Steubenville, USA

**Keywords:** single-use tape, quality improvement, cross-contamination, microbial contamination, operating room tape

## Abstract

Background: There are no existing practices or methods to ensure cleanliness, sterility, or prevent cross-contamination when it comes to common operating room (OR) tape. The authors hypothesized that adhesive tapes used by anesthesia providers in ORs and off-site surgical areas might be colonized by microorganisms and that culturing these tape rolls would reveal significant monomicrobial and polymicrobial contamination.

Material and Methods: The primary objective of this observational cohort study was to report and compare contamination rate including polymicrobial contamination rate between tape specimens collected from storage site and specimen from the ORs, off-sites, and after use on a patient. The outcome measures were the culture reports of the adhesive tapes. The authors then designed an intervention that integrated anesthesia providers' hand hygiene and maintenance of a barrier between the OR tapes and OR surfaces.

Results: The authors reported gross contamination and cross-contamination among the OR off-site tapes. The contamination rates reported for tapes from OR, off-site specimens, and patient specimens were 68.2%,63.2%, and 100%, respectively. The authors again cultured adhesive tapes after the intervention and reported improved outcomes.

Conclusions: The current quality improvement (QI) project identified the potential for OR tapes to serve as microbial vectors. The authors advocate environmental decontamination and anesthesia providers' hand hygiene in parallel as a part of routine anesthesia care in their practice and agree that the endotracheal tubes (ETTs) and orogastric or nasogastric tubes should be pre-packaged with single-use tape, which can be used for securing devices.

## Introduction

Medical tape is the most ubiquitous, portable, and essential piece of all of the medical equipment employed by anesthesia providers. One of the most common uses of adhesive tape in an operating room (OR) environment is to secure the endotracheal tubes (ETTs), orogastric tubes, nasogastric tubes, IV lines, arterial catheters, and central venous catheters. The tape is also used as a final layer to close surgical incisions and attach surgical drains and/or urinary catheters to patients. Also, a single roll may be manipulated by multiple healthcare workers, including but not limited to, physicians, nurses, and OR technicians. Often a medical tape is handled with ungloved hands [1]. Furthermore, there are no existing practices or methods to ensure cleanliness, sterility, or prevent cross-contamination as far as OR tape is concerned [2].

A contaminated adhesive placed in close contact with IV catheters and mucous membranes of the patient’s respiratory and urogenital tract for extended periods could contribute to local or systemic infections [1,2]. The potential for this increases manifold in immunocompromised patients and those with longer indwelling catheter times [3]. The OR tape may also perpetuate contamination and bioburden in the OR, especially involving the anesthesia workstation [4,5]. The contaminated anesthesia workstations are associated with 30-day postoperative infections impacting as many as 16% of surgical subjects [6]. In the authors’ setting, Durapore^TM^ tape (silk tape) is commonly used to secure artificial airways (ETT, supraglottic airways) and various drainage tubes, while Transpore^TM^ tape (transparent plastic tape) may be placed over the patient’s eyes after induction of anesthesia to prevent corneal ulceration and to secure IV catheters. It is quite common for OR tapes to be stocked together in the same compartment in an anesthesia cart.

The authors hypothesized that OR tapes might report significant monmicrobialo or polymicrobial contamination. The authors’ main goals were to identify OR tape as a microbial vector and report that current practices promote cross-contamination and polymicrobial seeding of microbes among rolls of tape. The authors designed a workflow to reduce the contamination rates of tape. They planned an observational study to measure if the change in workflow led to a measurable reduction in the rate of tape contamination. If the new workflow for handling OR tape was associated with a decrease in the contamination rates of OR tape, then the authors intended to promote the new workflow by making anesthesia providers, OR staff, and hospital administration aware of the issues identified and by releasing the results of this study. The authors planned a quality improvement (QI) project and integrated the above-mentioned objectives into various phases of this study.

## Materials and methods

**Figure 1:**
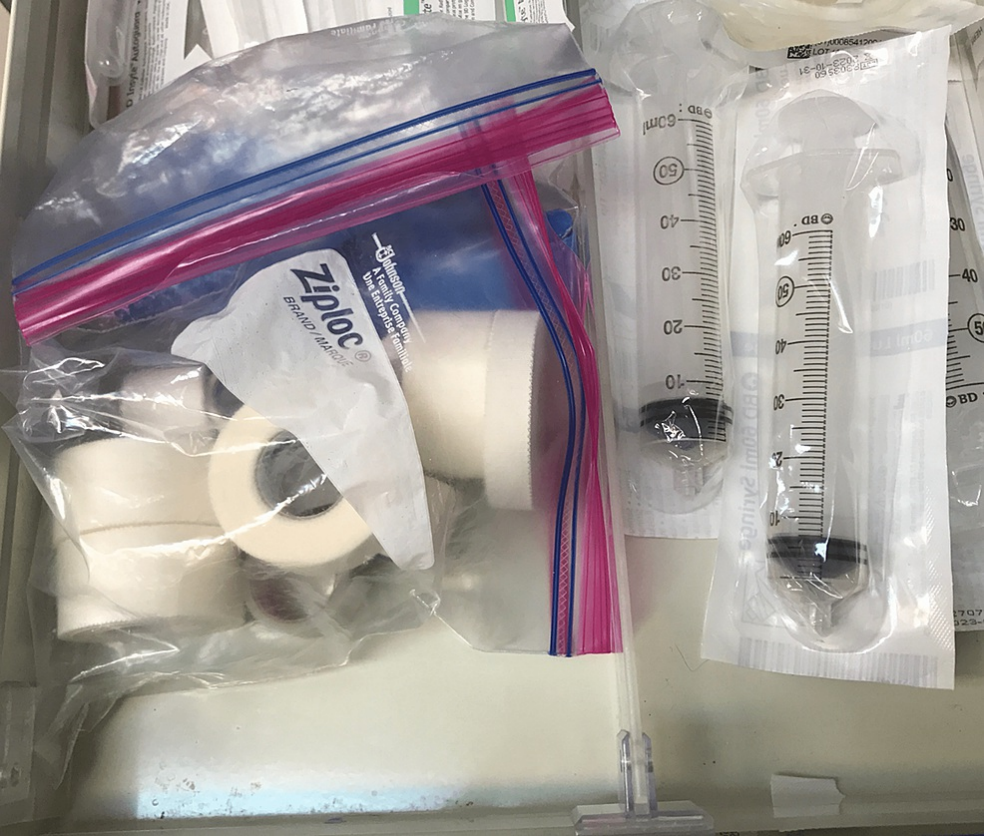
New and old (used on patient) tapes stored together in anesthesia cart in an OR OR: Operating room

The study setting was an urban academic hospital. In Phase I of our QI project, we planned a prospective observational study with the primary objective of reporting and comparing contamination rate, including the polymicrobial contamination rate of tape between negative control tape specimens collected from the storage site and active specimens from the ORs (Figure 1).

**Figure 2:**
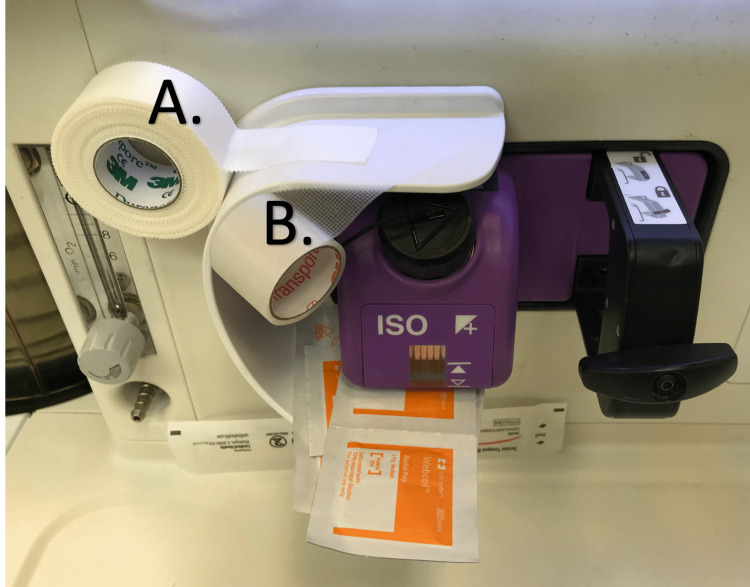
OR tapes (A) Durapore^TM ^tape used for securing ETTs, nasogastric, and orogastric tubes; (B) Transpore^TM^ tape used to cover the eyes after induction of anesthesia OR: Operating room; ETT: Endotracheal tubes

The secondary objectives were to report and compare contamination rates, including polymicrobial contamination rates between negative control tape specimens and off-sites, active test samples and patient samples (positive controls) from OR, all tape specimens obtained from OR and off-sites, between Durapore^TM^ and Transpore^TM^ tape and to report the most prevalent microbial flora on tape specimens from ORs and the off-sites (Figure 2).

The outcome measures were the culture reports of the Durapore^TM^ and Transpore^TM^ tapes, randomly obtained from the ORs, off-sites, and after use on patients. The investigators collected three types of specimens during Phase I: negative control samples, active test samples, and positive control samples. Negative control samples collected were 11 sets (Durapore^TM^ and Transpore^TM^) of new (out of the manufacturer's box) tape rolls that were randomly obtained from the storage site on the day the samples were collected from the OR. 18 sets of (Durapore^TM^and Transpore^TM^) new tape rolls were randomly obtained from the storage site on the day the samples were collected from the off-sites. All active test specimens were collected two hours prior to the time the OR was set to begin that day. This was done to ensure that no specimens were removed from a potentially contaminated OR. 58 tape rolls were collected from 11 randomly selected ORs and various off-site anesthesia locations. These tape rolls were taken from anesthesia carts, machines, and Mayo stands. Positive control collected 11 tape specimens (Durapore^TM^) that had been used to secure ETT or on the eyes (Transpore^TM^) of patients during the standard care of patients. These specimens were collected immediately upon removal from the patients, i.e., the tape was used to secure the ETT, and the rest of the tape was secured for testing.

After having collected and cultured the specimens, the authors analyzed the outcomes of Phase I of the project and then initiated Phase II of this QI study. For Phase II, we formulated a workflow to reduce the contamination rates of tape. The new workflow was implemented in nine ORs and nine off-sites, which were enrolled specifically as a part of Phase II. As part of Phase II, anesthesia providers were requested to strictly adhere to the sterile technique to procure the tape from the storage areas. The provider handling the tape had to wash hands between cases with antimicrobial soap and water and wear non-sterile gloves always when handling the tape. Then, upon transporting any tape from the anesthesia supply room to the OR, the tape was to be placed in a clear, single-use, 6" x 9" zip-lock transport bag. While placing the tape on a patient, the provider used non-sterile gloves and then returned the tape to the clear transport bag. The provider then discarded all unused tape after every case (single-use).

The authors then hypothesized that the new workflow to procure, use, and discard tape would lead to reduced contamination. The authors planned a second prospective observational study to measure the effect of the new workflow, with the primary objective to report and compare contamination rate, including polymicrobial contamination rate, post-intervention, of tapes between negative control tape specimens collected from storage site and positive control specimens (patient samples) from the ORs. The secondary objectives were to compare contamination rates of tapes between negative control tape specimens (storage site) and positive controls from off-site. The authors also compared contamination rates of all tapes between ORs and off-sites.

The outcome measures, post intervention, were culture reports of tapes from the enrolled ORs and off-sites and the storage site. Three types of samples collected for Phase 2 of the study were negative controls that included 10 sets (Durapore^TM^ and Transpore^TM^) of new tape rolls that were randomly obtained from the storage site. The positive controls included 10 tape specimens (Durapore^TM^) that were used to secure ETT and placed over the eyes (Transpore^TM^) of patients' post induction of anesthesia from the enrolled ORs. These specimens were collected after their use on the patients. Then, the third group of samples included positive controls that included 10 tape specimens (Durapore^TM^) that were used to secure ETT and placed over the eyes (Transpore^TM^) of patients post induction of anesthesia from the enrolled off-sites. The tape specimens were collected after their use on the patients.

Specimen collection, handling, and culture protocol

After obtaining approval from the hospital's institutional review board, the investigators proceeded with specimen collection, as outlined above. We based our collection protocol on one previously mentioned in a similar study by Redelmeier and Livesley [2]. Different types of samples were collected on different days. The investigators wore standard OR scrub attire, surgical masks, and OR caps during sample collection. The investigators then sanitized their hands with alcohol-based foam cleansers before entering any ORs, procedural areas, off-site, and the resident lounge. The sample collectors wore separate sterile gloves to collect each individual specimen. An investigator, who was a part of this study, was assigned to scrutinize the technique and ensure that the physicians collecting the tape adhered to sterile precautions and the proposed protocol. All rolls were meticulously handled in a sterile manner via the central hole and placed in a previously unopened a, clear, single-use, 6" x 9" zip-lock transport bag. Only a single roll of tape was placed in each specimen bag. The transport bags were then labeled with a corresponding letter and specimen number. Two investigators collected and labeled the specimens, while the third investigator closely observed to ensure that all sterile precautions were followed. 

Upon specimen collection from each site, the specimen bags were immediately transported to the microbiology lab to be processed. In the microbiology lab, approximately 4 inches of the tape was removed from each roll and cut into four 1-inch pieces using sterile gloves and sterile scissors. One piece from each end was placed into a sterile tube with 1 ml of sterile 0.8% saline, and the tubes were vortexed for 10 seconds. A 1 μl loop was used to inoculate a blood agar plate for semi-quantitation, and plates were incubated at 35°C for 18-24 hours. Each colony that grew was equated to 1,000 colony-forming units (CFU) per ml of liquid or 1,000 CFU per square inch of tape. Antibiotic sensitivity testing was performed based on the isolate identified, for e.g., Staphylococcus aureus for Methicillin resistance, Enterococcus species for Vancomycin resistance, and Enterobacteriaceae/Pseudomonas for Carbapenem resistance. 

Statistical analysis

The investigators expected that 70% of the tapes from OR and off-sites would report contamination, which concord with previous research on this subject [2]. With approximately 60 tapes in circulation in the ORs and off-sites enrolled in the study, for a two-sided T-test with alpha set at 0.05, a sample size of 51 tapes will provide 80% power to detect this effect. We examined 58 specimens to account for lost specimens, mishandling, etc. The contamination rates and polymicrobial colonization rates were expressed in percentages. The analysis employed to compare the results was a simple single-tailed, two-sample Student's T-test comparing the positive control specimens to the negative control specimens.

## Results

The negative control group reported no growth in all 22 specimens (11 Durapore^TM^ and 11 Transpore^TM^). As compared to the negative control group (0/22), the active specimen group reported a significantly higher contamination rate of 15/22 (68.2%) (p-value < 0.0001) (Table 1). The polymicrobial contamination rate of the active specimen group was also significantly higher (8/15, 53.3%) than the negative control group (p-value < 0.0001) (Table 1) . In order to compare the negative control group to the off-site specimen group, 36 tapes were collected for each group. As compared to the negative control group (0/36), the off-site specimen group reported a significantly higher contamination rate of 23/36 (63.9%) (p-value < 0.0001). The polymicrobial contamination rate of off-site specimen group reported being 41.7% (15/36), which was significantly higher than the negative control group (p-value < 0.0001) (Table 1). 

**Table 1:** Negative control (OR and off-sites) versus active test (OR and off-sites) samples The negative control samples were collected randomly from the storage site, and active control specimens were obtained from enrolled ORs and off-sites. The analysis employed to compare the results was simple single-tailed, two-sample, equal variance Student’s T-test comparing the positive control specimens to the negative control between storage site and OR tapes. OR: Operating room

Tape Types	OR		p-value	Off-Sites		p-value
	Negative Control (N = 22)	Active Specimens (N = 22)		Negative Control (N = 36)	Active Specimens (N = 36)	
Contaminated tapes (%)	0/22 (0%)	15/22 (68.2%)	<0.0001	0/36 (0%)	23/36 (63.9%)	<0.0001
Polymicrobial contamination (%)	0	8/15 (53.3%)	<0.0001	0	15/36 (41.7%)	<0.0001

The contamination rate of active specimen group (15/22, 68.2%), as compared to the contamination rate of positive control specimen (11/11, 100%), was not significantly different (p-value 0.07) (Table 2). The polymicrobial contamination rate of active specimen group (8/15, 53.3%), as compared to the polymicrobial contamination rate of positive control specimen (10/11, 90.9%), was also not significantly different (p-value 0.08) (Table 2). The contamination rate of all tape collected from the OR, that includes active tape specimens (n=11) and positive control specimen (n=22), was 78.8% (26/33), which was not significantly different from contamination rate of tape collected from off-sites, which was reported to be 63.9% (23/36) (p-value 0.17) (Table 2). The polymicrobial contamination rate of tape collected from the OR was reported to be 54.5% (8/33), which, as compared to the polymicrobial contamination rate of off-site specimens (15/36, 41.7%), was also not significantly different (p-value 0.17) (Table 2). 

**Table 2:** Active test (OR) samples versus positive control specimens from the OR and the comparison between all tape specimens from OR versus all tape specimens from off-site specimens. The analysis employed to compare the results was simple single-tailed, two-sample Student’s T-test comparing the active specimens to the positive control specimen. OR: Operating room

Tape Types	OR		p-value	OR (N=33)	Off-site (N=36)	p-value
	Active Specimen (N = 22)	Positive Control (N = 22)				
Contaminated tapes (%)	15/22 (68.2%)	11/11 (100%)	0.07	26/33 (78.8%)	23/36 (63.9%)	0.17
Polymicrobial contamination (%)	8/15 (53.3%)	10/11 (90.9%)	0.08	18/33 (54.5%)	15/36 (41.7%)	0.17

Further stratification of tapes samples obtained from off-sites reported the contamination rates of 100% for tapes collected from interventional radiology (IR) suites (3/3), endoscopy suites (6/6), and anesthesia provider fanny packs (4/4). On the other end of the spectrum, epidural carts located in the anesthesia workroom reported 0% (0/4) contamination rates. The other off-sites contamination rates were 42.9% (3/7) for obstetric suites, 50% (2/4) for regional block rooms, and 62.5% (5/8) for tapes collected from cardiac catheterization laboratories (Cath labs). The polymicrobial contamination rates reported were highest for provider waist packs at 75% (3/4), followed by IR suites at 66.7% (2/3), endoscopy suites at 66.7% (4/6), catheterization laboratory at 37.5% (3/8), obstetric suites at 28.6% (2/7), and regional nerve block suites at 25% (1/4) (Table 3).

**Table 3:** Contamination and polymicrobial contamination rates of various off-site locations IR: Interventional radiology; OR: Operating room

Off-sites sampling	Percentage of contaminated tapes	Percentage of polymicrobial contamination
IR suites	3/3 (100%)	2/3 (66.7%)
Providers’ waist pack	4/4 (100%)	3/4 (75.0%)
Block suites	2/4 (50.0%)	1/4 (25.0%)
Labor OR suites	3/7 (42.9%)	2/7 (28.6%)
Epidural carts	0/4 (0.0%)	0/4 (0.0%)
Catheterization laboratories	5/8 (62.5%)	3/8 (37.5%)
Endoscopy suites	6/6 (100%)	4/6 (66.7%)
Total specimens	23/36 (63.9%)	15/36 (41.7%)

As far as the material of tape is concerned, the contamination rate of 80% (32/40) reported for Durapore^TM^ tape was significantly higher than contamination rate of 58.6% (17/29) reported for Transpore^TM^ tape (p-value 0.05). However, the polymicrobial contamination rates of 55% (22/40) and 37.9% (11/29), for Transpore^TM^ and Durapore^TM^, respectively, did not report a significant difference (p-value 0.77).

The cultures in the OR reported a predominance of alpha-hemolytic Streptococci, S. mitis/oralis (54.5%, 18/33). The tape specimens from the ORs also reported polymicrobial contamination with Rothia mucilaginosa (21.2%, 7/33), Rothia decantirosa (3%, 1/33), S. salivarius/vesitibularis (21.2%, 7/33), Kocuria kristinae (9.1%, 3/33), and S. aureus (3%, 1/33). The cultures of tapes from the off-sites reported a predominance of alpha-hemolytic Streptococci (S. mitis/oralis: 41.7%, 15/36) and catalase-positive, coagulase-negative Staphylococci (S. epidermidis: 41.7%, 15/36) (Table 4). The off-sites also reported polymicrobial contamination with Streptococci (S. parasaguinis, S. salivarius/vesitibularis) and Staphylococci (S. hominis, S. epidermidis, and S. aureus). The authors’ facility has two regional anesthesia block rooms out of which culture of tapes from one block room reported polymicrobial growth with Methicillin-resistant Streptococcus hemolyticus. The tapes recovered from the anesthesia resident lounge and the fanny packs that the residents usually carry in the OR also reported 100-1,000 CFU/ml of polymicrobial growth of Methicillin-resistant S. hemolyticus (Table 5). In phase 2 (post-intervention phase), there was no microbial activity detected on the negative control group tapes. Furthermore, there was no contamination detected on the positive control tape specimens collected from the enrolled OR and off-sites (Table 6).

**Table 4:** Microbial flora of ORs OR: Operating room

Microorganism	Species	Percentage of tapes colonized *
Gram positive, alpha-hemolytic Streptococci	S. mitis/oralis	54.5% (18/33)
Gram positive, Coagulase-negative Stomatococcus	Rothia mucilaginosa	21.2% (7/33)
	Rothia dentocariosa	3% (1/33)
Gram positive, Catalase-negative Streptococci	S. parasanguinis	39.4% (13/33)
	S. salivarius/vesitibularis	21.2% (7/33)
Gram positive Micrococcus	Kocuria kristinae	9.1% (3/33)
Gram positive, Catalase-positive, coagulase negative Staphylococci	S. epidermidis	12.1% (4/33)
Gram positive, Catalase-positive, coagulase positive Staphylococci	S. Aureus	3% (1/33)
No growth		21.2% (7/33)

**Table 5:** Microbial flora of off-site locations

Microorganism	Species	Percentage of tapes colonized*
Gram positive, alpha-hemolytic Streptococci	S.mitis/ oralis	41.7% (15/36)
Gram positive, Catalase-negative Streptococci	S.parasanguinis	2.8% (1/36)
	S.salivarius/ vesitibularis	2.8% (1/36)
Gram positive, beta-hemolytic Streptococci	S.hemolyticus - MRSA	2.8% (1/36)
Gram positive, Catalase-positive, coagulase-negative Staphylococci	S.hominis	8.3% (3/36)
	S.warnerii	24.2% (8/33)
	S.hominis - MRSA	5.6% (2/36)
	S. epidermidis	41.7% (15/36)
Gram positive, Catalase positive, coagulase positive Staphylococci	S.aureus	5.6% (2/36)
No growth		36.1% (13/36)

**Table 6:** Post intervention: negative control (storage site) versus the active test (OR and off-sites) samples The negative control samples were collected randomly from the storage site, and positive control specimens were obtained from enrolled ORs and off-sites. The analysis employed to compare the results was simple single-tailed, two-sample, equal variance Student’s T-test comparing the positive control specimens to the negative control between storage site and OR tapes. OR: Operating room

Tape Types	OR		p-value	Off-Sites		p-value
	Negative Control (N = 20)	Positive Control (N = 20)		Negative Control (N = 36)	Active Specimen (N = 36)	
Contaminated tapes (%)	0/22 (0%)	0/20 (0%)	0.99	0/20 (0%)	0/20 (0%)	0.99
Polymicrobial contamination (%)	N/A	NA	NA	N/A	NA	NA

## Discussion

Our study highlights the significant bacterial growth found on commonly used OR tape. There was substantial bacterial growth reported for specimens collected as the positive control group and active test specimens. The organisms were diverse, and some tape rolls showed polymicrobial growth with the same genus of bacteria growing on multiple tapes, signifying rampant cross-contamination among the rolls. The cultures reported oral commensal and gram-positive, catalase-negative Staphylococci, and other multi-drug resistant bacteria contaminating OR tape rolls. These commensal bacteria are regarded as an emerging opportunistic pathogen increasingly associated with sepsis in immunocompromised hosts and prosthetic device infections such as in orthopedic implants, cardiac devices, or prosthetic heart valves [7-9]. Redelmeier and Livesley reported that 74% of the medical tape collected in one hospital was colonized by pathogenic bacteria [2]. Besides commensals, surgical tape is reported to be frequently contaminated with multidrug-resistant organisms [1,3]. There are multiple reports of infectious disease outbreaks in intensive care units and general hospital floors in which the source was traced back to medical tape [10-12]. Once the OR tape is circulated for patient care, it can become inoculated with bacteria within a matter of days and serve as an efficient vector promoting cross-contamination amongst patients [13]. The investigators reported that Durapore^TM^ tapes reported more florid contamination than Transpore^TM^ (80% vs 58.6%). This observation can be explained by the fact that the length of Durapore^TM^ tapes (10 yards) is more than that of the Transpore^TM^ tapes (1 yard). Thus, Durapore^TM^ tapes present in the OR for a longer period of time than Transpore^TM^ tapes. These observations are also corroborated by Berkowitz et al., who serially cultured the tape rolls on Days 1, 5, and 7 in an intensive care unit [3]. They reported no growth on the fresh rolls of tape. However, once the same rolls were circulated for patient care, the cultures yielded significant microbial growth. In a few days, the specimens reported being inoculated by single species of bacteria subsequently developed polymicrobial growth, signifying cross-contamination among the rolls of tape. Berkowitz et al. concluded that medical tape is an efficient vector, and contaminated tape is an efficient vector affecting multiple anesthesia providers and patients [3].

The perioperative bacterial seeding of intravenous catheters from contaminated OR tape is a cause for concern. Failure to adhere to appropriate hand hygiene after handling contaminated tape rolls may further increase the odds of introducing an infection into the bloodstream of patients undergoing surgery [4]. Irrefutable evidence implicates anesthesia providers’ hands and the surrounding environment as primary drivers of contamination in the OR [14,15]. Munoz-Price et al. reported that the probability of bacterial growth in an IV stopcock is a function of the number of bacterial colonies infesting the OR environment and the baseline hand contamination of anesthesia providers [16]. The potential for bacterial contamination during anesthesia care is corroborated by Birnbach et al. [17]. They employed a fluorescent marker during simulated airway management and observed a high degree of spread of the marker from the hands of the anesthesiologist to the face of the manikin, IV hubs, and other OR surfaces [17]. Both reports concluded that in addition to other measures to reduce surgical site infections (SSI), it is critical to decontaminate the OR, especially if a known source of infection is present. 

The current literature offers conclusive evidence that bacterial contamination of intravascular devices is associated with increased morbidity in a healthcare setting [2,18-20]. Loftus et al. reported a direct association and a higher probability of hospital-acquired infections (HAI) or SSI, leading to more extended hospital stays and increased 30-day postoperative morbidity and mortality due to bacterial seeding of IV stopcocks [6]. Loftus et al. summarized that suboptimal environmental decontamination and lack of hand hygiene was universal issue in all of the institutions in which their research was conducted [6]. The evidence at hand guided the authors to design a QI process that integrated anesthesia providers' hand hygiene and maintenance of a barrier between the OR tapes and OR surfaces to effect environmental decontamination. The authors measured the effect of this intervention with subsequent cultures reporting no growth in the positive controls and active test specimens. This prompted the authors to adopt this new process, geared towards workspace decontamination, as a part of routine anesthesia care in their practice. 

There are significant limitations to this study. The authors were focused on a single element of anesthesia practice that may contribute to the HAI or SSI during the intraoperative period. Many other factors are implicated in developing HAI or SSI, such as other reservoirs of infection in the OR environment, sub-optimal sterilization or barrier techniques, concurrent comorbidities, and reduced immunity, among other factors. The authors reported contamination rates of OR tapes but did not venture into the incidence of SSI in the cohort of patients that underwent surgery during the sample collection phase. The authors did not examine the presence of other microbes like viruses or fungi on the OR tapes. It was a single-center study, and the sample size in the study was small. The study reported the culture results of two brands of OR tapes, but other brands of tape may differ in their support of microbial colonization. The cultures of the tape rolls procured from the anesthesia storage area were negative for microbial activity. However, if the negative control specimens had reported contamination, the authors would have failed to launch the second phase of this QI project. This workflow paradigm led the authors to conclude that the QI workflow they designed is not a long-term solution to reduce the incidence of HAI or SSI due to OR tape rolls during the intraoperative period. The authors unanimously agreed that the ETT, nasogastric, or orogastric tubes should be pre-packaged with single-use tape, which can be used for securing devices. A large proportion (60% or more) of SSIs are preventable, and payers and insurers may no longer reimburse the incremental readmission cost [21-23].

## Conclusions

In conclusion, the current QI project identified the potential for OR tapes to serve as microbial vectors, measured the contamination rates of OR tapes, and reported effusive contamination of the OR tapes with rampant cross-contamination among tape rolls. The authors unanimously agree that passive neglect of OR bioburden may conceivably offset the advantage of meticulous hand hygiene and other more publicized measures to reduce HAI or SSI. The authors designed the workflow integrating environmental decontamination and anesthesia providers' hand hygiene in parallel and reported improved outcomes with the new approach. The observations of the current study convinced the authors to recommend against storing OR tape in anesthesia carts. The authors further advocate for single-use OR tape for patient care. The authors also advise strict adherence to hand hygiene practices, especially while handling tape in the healthcare setting. Healthcare providers need to develop a heightened awareness of the intraoperative transmission of potentially pathogenic organisms. The physicians should be invested in continued research and critical appraisal of factors involved in SSI/ HAI and implement effective preventive measures. The ongoing risks of HAI and SSI are also prompting hospitals to seek new methods for reducing cross-contamination, and the use of pre-packaged single-use tape for securing devices such as ETT, nasogastric, or orogastric tubes handled by anesthesia providers, could be a simple yet appealing solution.
